# Bone marrow‐derived mesenchymal stem cells promote invasiveness and transendothelial migration of osteosarcoma cells via a mesenchymal to amoeboid transition

**DOI:** 10.1002/1878-0261.12189

**Published:** 2018-03-31

**Authors:** Laura Pietrovito, Angela Leo, Valentina Gori, Matteo Lulli, Matteo Parri, Valentina Becherucci, Luisa Piccini, Franco Bambi, Maria Letizia Taddei, Paola Chiarugi

**Affiliations:** ^1^ Department of Experimental and Clinical Biomedical Sciences University of Florence Italy; ^2^ Transfusion Medicine and Cell Therapy Meyer Children's Hospital Florence Italy; ^3^ Department of Experimental and Clinical Medicine University of Florence Italy

**Keywords:** bone marrow‐derived mesenchymal stem cells, cytokines, osteosarcoma, transendothelial migration, tumour plasticity

## Abstract

There is growing evidence to suggest that bone marrow‐derived mesenchymal stem cells (BM‐MSCs) are key players in tumour stroma. Here, we investigated the cross‐talk between BM‐MSCs and osteosarcoma (OS) cells. We revealed a strong tropism of BM‐MSCs towards these tumour cells and identified monocyte chemoattractant protein (MCP)‐1, growth‐regulated oncogene (GRO)‐α and transforming growth factor (TGF)‐β1 as pivotal factors for BM‐MSC chemotaxis. Once in contact with OS cells, BM‐MSCs trans‐differentiate into cancer‐associated fibroblasts, further increasing MCP‐1, GRO‐α, interleukin (IL)‐6 and IL‐8 levels in the tumour microenvironment. These cytokines promote mesenchymal to amoeboid transition (MAT), driven by activation of the small GTPase RhoA, in OS cells, as illustrated by the *in vitro* assay and live imaging. The outcome is a significant increase of aggressiveness in OS cells in terms of motility, invasiveness and transendothelial migration. In keeping with their enhanced transendothelial migration abilities, OS cells stimulated by BM‐MSCs also sustain migration, invasion and formation of the *in vitro* capillary network of endothelial cells. Thus, BM‐MSC recruitment to the OS site and the consequent cytokine‐induced MAT are crucial events in OS malignancy.

**Abbreviations**
(TGF)‐β1: transforming growth factor
ANG: angiopoietin
BM‐MSCs: bone marrow‐derived mesenchymal stem cells
BSA: bovine serum albumin
CAFs: cancer‐associated fibroblasts
CAMs: cancer‐associated macrophages
CM: conditioned medium
DMEM: Dulbecco's modified Eagle's medium
ECM: extracellular matrix
EMT: epithelial‐to‐mesenchymal transition
FBS: fetal bovine serum
GRO: growth‐regulated oncogene
HUVECs: human umbilical vein endothelial cells
IL: interleukin
MAT: mesenchymal to amoeboid transition
MCP: monocyte chemoattractant protein
MLC: myosin light chain
MMPs: matrix metalloproteinases
NG2: neural/glial antigen 2
OPG: osteoprotegerin
OS: osteosarcoma
PDGF‐BB: platelet‐derived growth factor BB
P‐MLC: active form of myosin light chain
SDF‐1: stromal cell‐derived factor 1
TβR blk: TGF‐β receptor blocker
VEGF: vascular endothelial growth factor

## 1 Introduction

Osteosarcoma (OS) is an aggressive primary malignant bone tumour accounting for ~ 60% of all bone sarcomas, affecting mainly paediatric patients. It is characterized by early metastasis, primarily to the lung, and tumour relapse (Gibbs *et al*., 2005; Meyers *et al*., 2011). The clinical outcome for OS patients with metastatic or relapsed OS has remained unchanged over the past 30 years, underlining the need for new therapeutic strategies (Kansara *et al*., 2014).

Cancer cell plasticity is a key prerequisite for ensuring metastatic dissemination of the tumour, and describes the ability of cancer cells to change their migration style in response to environmental conditions (Odenthal *et al*., 2016). Tumour cells can move as individual cells or collective groups. Additionally, invasive single cell migration can be divided into mesenchymal style, characterized by elongated cell morphology and strongly dependent on extracellular matrix (ECM) proteolysis, and amoeboid migration, showing a rounded cell morphology and independence from ECM degradation and adhesion (Friedl and Alexander, 2011; Sanz‐Moreno *et al*., 2008). During tumour progression, cancer cells can switch between different motility styles to allow the escape of tumour cells from the primary site and subsequent dissemination to distant organs (Friedl and Wolf, 2003). The epithelial‐to‐mesenchymal transition (EMT) allows the trans‐differentiation of a cell with epithelial features into a motile mesenchymal cell. This is a complex transcriptional programme that involves loss of cell–cell junctions, adhesions to ECM and cell polarity. These events are associated with achievement of migratory and invasive abilities. In addition, a cell moving with a mesenchymal style can undergo a transition into amoeboid style motility, a process known as mesenchymal‐to‐amoeboid transition (MAT; Friedl and Wolf, 2003). MAT is characterized by extensive changes in cell morphology and cytoskeleton organization. Due to low reliance on cell adhesion and ECM proteolysis, amoeboid motility is significantly faster and less energy‐consuming compared with the mesenchymal mode. Moreover, high cytoskeleton contractility may confer the advantages of promoting intravasation and metastatic dissemination of tumour cells (Friedl and Wolf, 2010; Sanz‐Moreno *et al*., 2008).

Growing evidence indicates that bone marrow‐derived mesenchymal stem cells (BM‐MSCs) represent critical actors in the tumour microenvironment (Barcellos‐de‐Souza *et al*., 2013; Cuiffo and Karnoub, 2012; Gwendal and Paula, 2016). Several studies have demonstrated that inflammatory cytokines and growth factors produced by different primary epithelial cancers can recruit BM‐MSCs (Barcellos‐de‐Souza *et al*., 2016; Cuiffo and Karnoub, 2012; Spaeth *et al*., 2008; Tsukamoto *et al*., 2012; Xu *et al*., 2009). Once recruited into the tumour stroma, BM‐MSCs may sustain tumour growth and progression in multiple ways. Indeed, BM‐MSCs may directly affect the malignancy cancer cells (Karnoub *et al*., 2007; Xu *et al*., 2009), support tumour angiogenesis (Au *et al*., 2008; Suzuki *et al*., 2011), differentiate into other pro‐tumourigenic stromal cells, such as cancer‐associated fibroblasts (CAFs) and cancer‐associated macrophages (CAMs) (Barcellos‐de‐Souza *et al*., 2016; Mishra *et al*., 2008), and act as immune‐modulators to suppress both innate and adaptive immune responses against cancer (Nauta and Fibbe, 2007; Sotiropoulou and Papamichail, 2007). However, there is also evidence indicating an anti‐tumoural activity of BM‐MSCs (Attar‐Schneider *et al*., 2016; Lee *et al*., 2013; Qiao *et al*., 2008). Indeed, the role of BM‐MSCs in promoting tumourigenesis is still controversial and warrants further studies.

Here, we evaluated the *in vitro* effects of the cross‐talk between BM‐MSCs and OS cells on tumour malignancy. We have used the conditioned medium (CM) derived from either BM‐MSCs or three different OS cell lines: SaOS‐2, MG‐63 and HOS. These cells differ in chromosomal alterations, proliferation rate, invasion behaviour and expression profiles of cytokines, growth factors and matrix proteins (Lauvrak *et al*., 2013; Mohseny *et al*., 2011). We proved that BM‐MSCs are efficiently recruited by monocyte chemoattractant protein (MCP)‐1, growth‐regulated oncogene (GRO)‐α and transforming growth factor (TGF)‐β1 produced by OS cells. Once in contact with tumour cells, BM‐MSCs enhance the levels of GRO‐α, MCP‐1, interleukin (IL)‐6 and IL‐8 in the tumour microenvironment. This pattern of cytokines is crucial to promote a MAT in OS cells, with a consequent increase in their motility, invasiveness and transendothelial migration. Moreover, the cross‐talk between BM‐MSCs and OS cells is crucial to promote a strong activation of endothelial cells. Accordingly, understanding whether this interplay is critical for *in vivo* tumour progression could offer an array of alternative targets to test in preclinical models for the impairment of OS metastatic dissemination.

## 2 Materials and methods

### 2.1 Antibodies and reagents

The following antibodies were used for western blot analysis: CollagenI‐α1 (NB600‐408, rabbit; Novus Biologicals, Littleton, CO, USA), α‐SMA (A2547, mouse), Rac1 (07‐1464, rabbit) and tubulin (T5168, mouse) from Sigma‐Aldrich (St. Louis, MO, USA) and RhoA (sc‐418, mouse, Santa Cruz Biotechnology, Santa Cruz, CA, USA). Secondary antibodies to the appropriate species were from Santa Cruz Biotechnology.

For the immunofluorescence experiments, FITC‐phalloidin (F432, Molecular Probes, Eugene, OR, USA), anti‐P‐MLC (Ser 19) antibodies (3671, rabbit, Cell Signaling, Danvers, MA, USA) and secondary antibodies conjugated with AlexaFluor 488 (A‐11034, Life Technologies Invitrogen, Carlsbad, CA, USA) were used.

For the migration experiments, blocking antibodies were used against: CXCR4 (555971, BD Bioscience, Franklin Lakes, NJ, USA), MCP‐1 (555055, BD Biosciences), IL‐6 (mabg‐hil6‐3, InvivoGen, San Diego, CA, USA) and IL‐8 (MAB208‐100, R&D System, Minneapolis, MN, USA). As control antibody, we used normal mouse IgG control (sc‐2025, Santa Cruz Biotechnology). SB225002 [(*N*‐(2‐hydroxy‐4‐nitrophenyl)‐*N*′‐(2‐bromophenyl)urea, 559405] and Ilomastat (GM 6001, 364205) were from Merck Millipore (Billerica, MS, USA). TGF‐β1R blocker (TβR blk, p17) was developed by Digna Biotech (Pamplona, Spain) as previously described (Barcellos‐de‐Souza *et al*., 2016).

Matrigel™ Basement Membrane Matrix (356234) was from BD Biosciences. Rho Activator (calpeptin, CN01) was from Cytoskeleton, Inc. (Denver, CO, USA); GST‐Rhotekin (14‐662) and recombinant human TNF‐α (300‐01A) were from Peprotech (Peprotech Inc, Rocky Hill, NJ, USA) and CellTrace™ carboxyfluorescein succinimidyl ester (CFSE, C34554) was from Life Technologies.

### 2.2 Isolation and culture cells

Human OS cell lines (SaOS‐2, MG‐63 and HOS) and human umbilical vein endothelial cells (HUVECs) were purchased from Sigma Aldrich (ECACC). Tumour cells were maintained in Dulbecco's modified Eagle's medium (DMEM) high glucose with 2 mm l‐glutamine (Euroclone, Milan, Italy) supplemented with fetal bovine serum (FBS; 10% v/v, Euroclone) and penicillin/streptomicin (P/S, 1% v/v, Euroclone), in cell culture flasks until 70–80% cell confluence. HUVECs were cultured in complete endothelial cell growth medium (EBM‐2 Basal Medium, Lonza, Basel, Switzerland) plus the SingleQuots Kit, supplemented with 2 mm l‐glutamine, P/S (1% v/v) and FBS (10% v/v). Human BM‐MSCs used in this study were isolated from healthy donors and characterized by Dr Bambi's Unit (AOU Meyer Hospital, Florence, Italy) as previously reported (Barcellos‐de‐Souza *et al*., 2016). BM‐MSCs were cultured in DMEM low glucose (Euroclone) containing FBS (10% v/v), 2 mm l‐glutamine and P/S (1% v/v) and used between passages 2 and 7.

### 2.3 Preparation of conditioned media (CM)

OS CM were obtained from 1 × 10^6^ tumour cells maintained in low glucose media deprived of serum (St Med) for 48 h at 37 °C in 5% CO_2_ humidified atmosphere. CM derived from untreated (BM‐MSCs ^St^) and conditioned MSCs (BM‐MSCs ^OS^) were obtained from BM‐MSCs grown to sub‐confluence and serum‐starved in low glucose media or stimulated with CM from OS cells for 48 h. Media were then removed and replaced with St Med (low glucose) for an additional 24 h. CM derived from the different experimental conditions were harvested, clarified by centrifugation and frozen at −80 °C until use.

### 2.4 *In vitro* migration assays

Migration assays were performed in Boyden Chamber with 8‐μm pore size filters (CC3422, Costar™, Corning, NY, USA). In BM‐MSC chemotaxis assays, 2.5 × 10^4^ cells were serum‐starved for 24 h and allowed to migrate overnight toward CM from SaOS‐2, MG‐63 and HOS cells. Untreated cells (St Med) were used as control. Migrating cells were fixed, stained and counted in four randomly chosen fields (10×) in bright field. In chemotaxis experiments with inhibitors, BM‐MSCs were starved overnight in the presence or absence of 20 μg·mL^−1^ anti‐ CXCR4 blocking antibodies, 200 nm SB225002 and 100 μg·mL^−1^ TβR blk. Anti‐MCP‐1 neutralizing antibodies 5 μg·mL^−1^ were added to CM 1 h before performing the assays. Migration assays of HOS cells were performed by treating 3.5 × 10^5^ tumour cells with CM BM‐MSCs ^St^ or CM BM‐MSCs ^OS^ for 24 h. St Med was used as control. Then, 5 × 10^4^ HOS cells were allowed to migrate for 6 h toward complete medium (FBS 10%). Invasion assays were achieved by covering the upper compartment of the Boyden chamber with 50 μg·cm^−2^ of reconstituted Matrigel. OS cells were treated with CM from starved or tumour‐activated BM‐MSCs for 36 h. Then 5 × 10^4^ HOS and 1 × 10^5^ SaOS‐2 or MG‐63 were allowed to migrate toward complete medium (10% FBS) for 5 h, overnight or 24 h, respectively. Transendothelial migration was performed with OS cells treated as above and stained with CFSE. Tumour cells (3 × 10^4^ HOS and 8 × 10^4^ MG‐63 and SaOS‐2) were seeded onto 5 × 10^4^ HUVECs activated with 10 ng·mL^−1^ TNF‐α and allowed to migrate toward 500 μL of complete medium (HOS for 5 h, MG‐63 and SaOS‐2 for 16 h). In invasion and transendothelial migration assays with inhibitors, conditioned HOS cells were treated or not treated with neutralizing antibodies against IL‐6 (5 μg·mL^−1^), IL‐8 (10 μg·mL^−1^), MCP‐1 (10 μg·mL^−1^) and SB225002 (200 nm). To evaluate MMP dependence, OS cells treated or not treated with BM‐MSCs CM were incubated overnight with 50 μm Ilomastat. The number of migrating cells was determined by counting in four randomly chosen fields in an inverted optical or fluorescent microscope for invasion and transendothelial migration, respectively. Recruitment assays of HUVECs were performed allowing migration or invasion of 5 × 10^4^ cells for 6 h toward CM HOS ^St^ and CM HOS ^BM‐MSCs^. St Med was used as negative control. Representative images of migration assays are reported in Supporting Information.

### 2.5 Western blotting

Cells were lysated in RIPA buffer and 5–20 μg of total proteins were loaded on precast SDS/PAGE gels (Bio‐Rad) as previously described (Taddei *et al*., 2014).

### 2.6 Collagen contraction assay

BM‐MSCs 1.5 × 10^5^ maintained for 24 h in St Med or HOS CM were harvested and resuspended in a DMEM solution containing 1 mg·mL^−1^ Collagen A (L7220, Merck Millipore) as previously reported (Barcellos‐de‐Souza *et al*., 2016). The area of each gel (number of pixels) was determined using imagej.

### 2.7 Gelatin zymography

CM derived from untreated or conditioned MSCs was collected, centrifuged and concentrated 10‐fold with Amicon Ultra 4 mL centrifugal filter (UFC800324, Merck Millipore). Gelatin zymography was performed as previously described (Taddei *et al*., 2014).

### 2.8 Pull‐down assay

RhoA and Rac1 activity were determined as previously reported (Taddei *et al*., 2014).

### 2.9 Confocal analysis

MG‐63 treated with CM from tumour‐activated BM‐MSCs CM was fixed in *p*‐formaldehyde (4% v/v in PBS) for 20 min, permeabilized in Triton X‐100 (0.5 v/v in PBS) for 5 min, then washed twice with bovine serum albumin (BSA; 1% v/v) and FBS (5% v/v in PBS) solution and incubated overnight at 4 °C with primary antibodies against P‐MLC (1 : 100). After two washes with PBS, cells were incubated with anti‐rabbit AlexaFluor 488 antibodies (1 : 1000) and FITC‐phalloidin for 1 h at room temperature in the dark. As positive control, we used 1 U·mL^−1^ Calpeptin, a RhoA activator. The coverslips were mounted in Gel Mount™ Aqueous Mounting Medium (Sigma‐Aldrich). A Nikon Eclipse TE2000‐U (Nikon, Tokyo, Japan) confocal microscope was used for data acquisition.

### 2.10 Flow cytometry

Staining of BM‐MSCs cultivated in St Med or treated with CM from OS cells for 48 h was performed as previously described (Barcellos‐de‐Souza *et al*., 2016).

### 2.11 ELISA and cytokine antibody array

Cytokine concentration in CM from MG‐63 cells and from BM‐MSCs was determined by ELISA single kits for IL‐1 alpha (EH2IL1A), IL‐6 (EH2IL6), IL‐8 (EH2IL8), IL‐17 (EH2IL17), GRO‐α (EHCXCL1), MCP‐1 (KHC1011), PDGF‐BB (EHCSRP2), TGF‐β1 (EHTGFBI) and TNF‐α (EH3TNFA), according to the manufacturer's instructions (Invitrogen). The qualitative analysis of cytokines and growth factors produced by BM‐MSCs was performed in CM obtained from 7.5 × 10^5^ cells grown in St Med or stimulated with CM from HOS cells for 48 h. Cells were then starved for a further 24 h and CM was collected, clarified by centrifugation and analysed with Human Cytokine Antibody Array C5 (AAH‐CYT‐5, RayBiotech, Aachen, Germany) according to the manufacturer's protocol. Membranes were developed with streptavidin‐HRP chemiluminescence reaction and then exposed to X‐ray film. Pixel densities of detectable spots were calculated using imagej software. The intensity of each spot was normalized to the intensities obtained using positive antibody array controls and subtracted from the related background. Expression values higher than 10 000 arbitrary units were set to identify a cut‐off for cytokines to be considered. The same type of analysis was performed in CM obtained from HOS cells stimulated or not stimulated with CM from tumour‐activated BM‐MSCs to determine the relative amounts of pro‐angiogenic factors.

### 2.12 RT‐qPCR

Extraction and retrotranscription of total RNA were performed as previously described [25]. Primers: IL‐8: 5′‐CTGGCCGTGGCTCTCTTG‐3′ (forward), 5′‐TTAGCACTCCTTGGCAAAACTG‐3′ (reverse); VEGF‐A: 5′‐TACCTCCACCATGCCAAGTG‐3′ (forward), 5′‐ATGATTCTGCCCTCCTCCTTC‐3′ (reverse). Data were normalized to those obtained with β‐2 microglobulin: 5′‐AGTATGCCTGCCGTGTGAAC‐3′ (forward), 5′‐GCGGCATCTTCACAAACCTCCA‐3′ (reverse). Results are the mean ± SD of three different experiments.

### 2.13 Capillary morphogenesis assay

HUVECs 2 × 10^4^ were starved overnight and added to the Matrigel‐coated well of a 96‐well plate in 200 μL of CM from HOS ^St^ or HOS ^BM‐MSCs^. The wells were photographed in three randomly chosen fields after 6 h, with an inverted microscope (Leitz DM IRB) equipped with CCD optics and a digital analysis system. Results were quantified by counting number of junctions for each field (10×).

### 2.14 Cell migration in three‐dimensional collagen matrices

Reconstruction by time‐lapse video microscopy and confocal microscopy was performed on MG‐63 cells. Subconfluent MG‐63 cells treated or not treated with CM from tumour‐activated BM‐MSCs were detached by EDTA (2 mm), washed, incorporated into three‐dimensional collagen lattice (1.67 mg·mL^−1^; native dermal bovine type I collagen; RD Systems) and monitored by time‐lapse video microscopy (Bröcker, 2004; Friedl, 2004). For three‐dimensional time‐lapse confocal microscopy (Leica‐SP5 system), cells within the lattice were labelled by CFSE (360 ng·mL^−1^), scanned for 12 h at 3‐min time intervals for simultaneous fluorescence and back scatter signal (reflection), and reconstructed. Three‐dimensional motility of cells is shown by time lapse of xyzt analysis (three‐dimensional analysis over time).

### 2.15 Statistical analysis

One‐way anova followed by a Bonferroni comparison test and Student's *t*‐test (two‐tailed) were used to determine statistical significance with a *P*‐value threshold set at < 0.05.

## 3 Results

### 3.1 BM‐MSCs disclose significant tropism for CM from OS cells and strongly affect the metastatic potential of cancer cells

**Figure 1:**
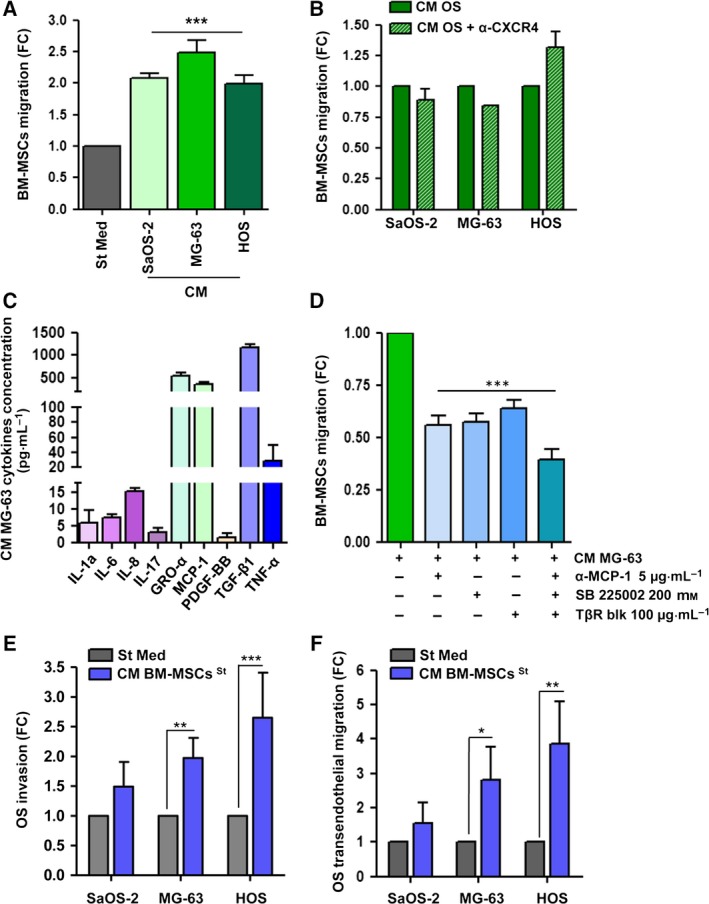
BM‐MSCs migrate toward CM from OS cells and affect the invasive behaviour of tumour cells. (A) BM‐MSCs were allowed to migrate overnight toward CM from OS cells (SaOS‐2, MG‐63 and HOS). Starvation medium (St Med) was used control. Results are expressed as mean ± SEM of five biological replicates; *** *P *<* *0.001 vs. St Med. (B) BM‐MSCs starved for 24 h in the presence or absence of neutralizing antibodies against CXCR4 (20 μg·mL^−1^) were allowed to migrate toward CM from OS cells. Results are expressed as mean ± SEM of three biological replicates. (C) ELISA of cytokines and growth factors in CM derived from MG‐63 starved for 48 h (mean ± SD,* n *=* *3 technical replicates). (D) BM‐MSC migration toward CM from MG‐63 with neutralizing antibodies against MCP‐1 (5 μg·mL^−1^, α‐MCP‐1), blocking of GRO‐α receptor (200 nm, SB 225002) and TGF‐β1 receptor (100 μg·mL^−1^, TβR blk). Results are presented as mean ± SEM of three biological replicates; *** *P *<* *0.001 vs. CM MG‐63. (E) OS cells were mantained for 48 h in St Med or CM obtained from BM‐MSCs starved for 48 h. Cells were then allowed to invade or (F) transmigrate toward complete medium (FBS 10%). Results are presented as mean ± SEM of three biological replicates; * *P *<* *0.05; ** *P *<* *0.01; *** *P *<* *0.005 vs. St Med.

In agreement with data from the literature showing strong homing properties of BM‐MSCs for several primary tumours, including OS (Barcellos‐de‐Souza *et al*., 2016; Tsukamoto *et al*., 2012; Xu *et al*., 2009; Yu *et al*., 2015), we demonstrated a significant chemotaxis of BM‐MSCs toward CM obtained from three different OS cell lines (SaOS‐2, MG‐63 and HOS), with a 2‐ to 2.5‐fold increase in migration when compared with control (St Med, Figs 1A and S1A). It has recently been reported that stromal cell‐derived factor 1 (SDF‐1 or CXCL12) is crucial to promote BM‐MSCs homing to CM from SaOS‐2 cells (Xu *et al*., 2009). However, we excluded the involvement of the CXCR4/SDF‐1 axis in the OS‐dependent recruitment of BM‐MSCs by performing chemotaxis experiments with blocking antibodies against CXCR4 (Figs 1B, S1B and S1C). Accordingly, to identify the soluble factors involved in the BM‐MSC chemotaxis toward OS cells, CM derived from MG‐63, the cell line that showed the highest chemoattractant abilities, was analysed by ELISA. We quantified established inflammatory cytokines and growth factors that have been shown to be involved in BM‐MSC migration in other cancer models (Fig. 1C; Barcellos‐de‐Souza *et al*., 2013; Spaeth *et al*., 2008). Among the cytokines produced, we focused our attention on those with the highest levels of secretion: GRO‐α (542 pg·mL^−1^ ± 70), MCP‐1 (359.9 pg·mL^−1^ ± 65) and TGF‐β1 (1161.7 pg·mL^−1^ ± 81), a pattern of cytokine expression common to other OS cell lines, as reported in several studies (Chen *et al*., 2015; Giner *et al*., 2015; Liu *et al*., 2017; Tu *et al*., 2014). To validate the role of these chemokines in BM‐MSC recruitment, we performed transwell migration assays with the following inhibitors: neutralizing antibodies against MCP‐1 (α‐MCP‐1), a TGF‐β receptor blocker (TβR blk) and a pharmacological inhibitor of the GRO‐α receptor, SB225002 (Boppana *et al*., 2014). As shown in Fig. 1D (and in Fig. S1D), the treatment of MG‐63‐derived CM with anti‐MCP‐1 antibodies, or the incubation of BM‐MSCs with SB225002 and TβR blk, significantly reduces BM‐MSC migration by about 50% compared with untreated MG‐63‐derived CM. Moreover, the combined treatment of all the compounds further reinforces this effect, confirming the key role of these cytokines in BM‐MSC chemotaxis toward CM from tumour cells. As control for MCP‐1 blocking antibody, we used normal mouse IgG, which did not significantly affect BM‐MSC chemotaxis toward CM from MG‐63 (Fig. S1E). The effects of the treatments on cell viability were checked by AnnV/PI staining (Fig. S1F).

Almost 50% of OS patients develop lung metastasis, the foremost cause of death for this tumour (Kansara *et al*., 2014; Meyers *et al*., 2011). Here, we investigated whether the conditioning of OS cells by BM‐MSCs could affect the metastatic potential of cancer cells. OS cells were incubated for 48 h with CM derived from BM‐MSCs, and their invasion and intravasation abilities were analysed by transwell migration assays. We found that the treatment of OS cells with CM from BM‐MSCs significantly increases tumour cell migration through either a Matrigel‐coated membrane (Figs 1E and S2A) or a monolayer of human umbilical vein endothelial cells (HUVECs, Figs 1F and S2B), thus suggesting the positive role of BM‐MSCs in invasive properties of OS cells.

### 3.2 BM‐MSCs acquire a CAF‐like phenotype upon contact with tumour cells

**Figure 2:**
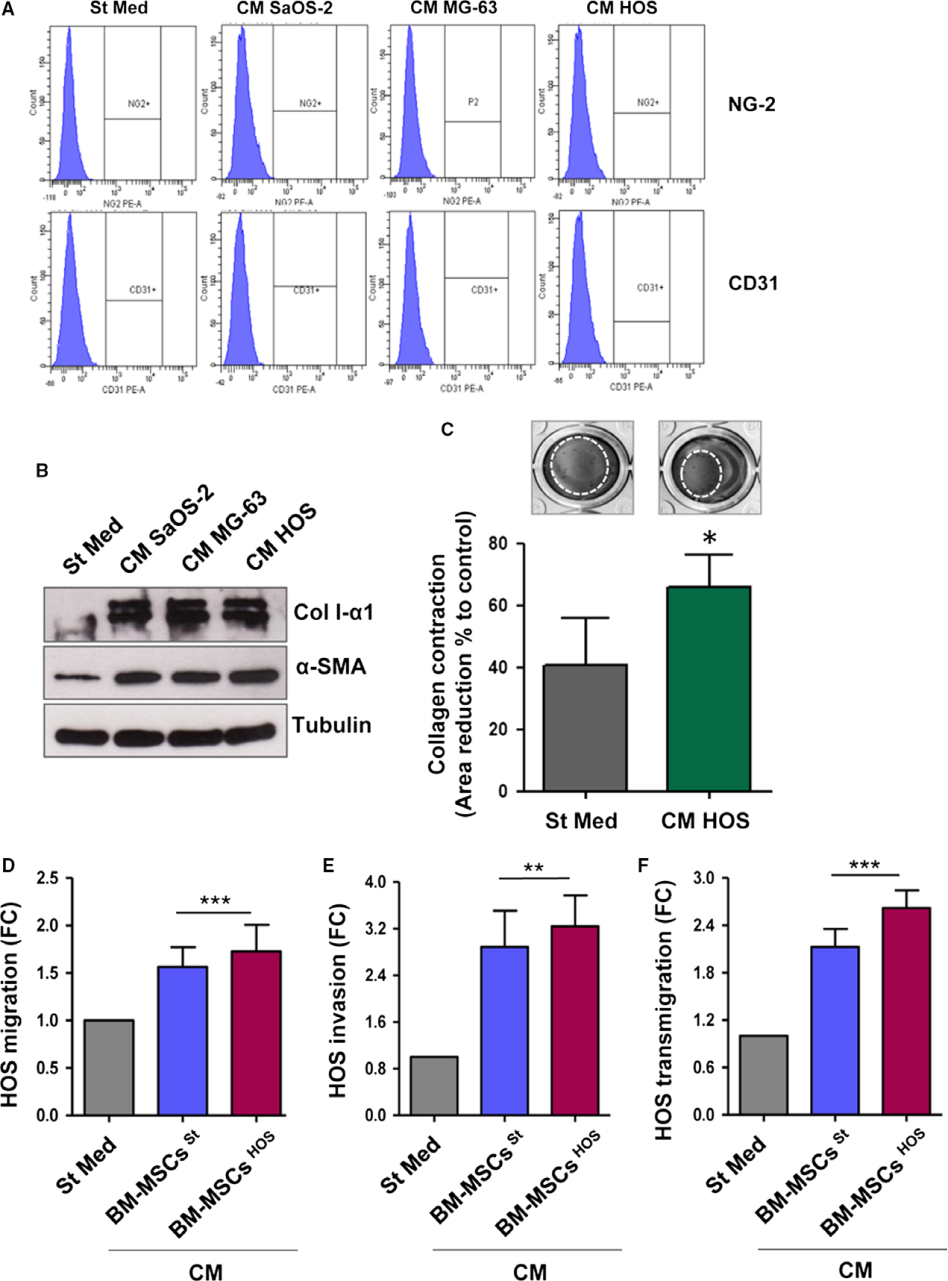
CM derived from OS cells stimulates the BM‐MSCs trans‐differentiation into CAF‐like cells. (A) FACS analysis of NG‐2 and CD31 expression in BM‐MSCs treated for 48 h with CM OS cells. (B) BM‐MSCs were stimulated for 48 h with CM from OS cells and α‐SMA and Col I‐α1 expression was assessed by immunoblot analysis. Results are representative of four biological replicates. (C) Collagen contraction assay of BM‐MSCs treated for 24 h with St Med or HOS CM. Data are expressed as percentages of the relative area of collagen disc following contraction in comparison with an empty well (mean ± SD,* n *=* *3 biological replicates performed in duplicate). * *P *<* *0.05 vs. St Med. (D) Migration assay of HOS cells stimulated for 48 h with CM derived from BM‐MSCs previously activated or not activated by tumour cells (CM BM‐MSCs ^St/^ ^HOS^). Cells were allowed to migrate toward complete medium (FBS 10%). Untreated cells (St Med) were used as control. Results are the mean ± SEM of three biological replicates. *** *P *<* *0.001 vs. St Med. (E) Invasion and transmigration (F) assays of HOS cells treated as in (D) (mean ± SD,* n *=* *3 independent experiments). ** *P *<* *0.005 vs. St Med; *** *P *<* *0.001 vs. St Med.

Once engrafted into the tumour microenvironment, BM‐MSCs establish a cross‐talk with cancer cells which may promote the trans‐differentiation of BM‐MSCs towards different tumour stromal cells, such as pericytes, endothelial cells and cancer‐associated fibroblasts (CAFs; Barcellos‐de‐Souza *et al*., 2016; Mishra *et al*., 2008). To determine whether soluble factors released by OS cells could stimulate a trans‐differentiation of BM‐MSCs into vascular‐like cells, BM‐MSCs were incubated for 48 h with CM derived from OS cells and analysed by flow cytometry for the expression of CD31 and neural/glial antigen 2 (NG2), established markers of endothelial cells and pericytes, respectively. As shown in Fig. 2A, following conditioning with CM from OS cells, the levels of CD31 and NG2 in BM‐MSCs are unaffected. To analyse a possible trans‐differentiation of BM‐MSCs into CAF‐like cells, we assessed the expression of α‐SMA and collagen I‐α1 in tumour‐activated BM‐MSCs by western blot analysis. As shown in Fig. 2B, the levels of both proteins significantly increase after CM from OS exposure. In keeping, the conditioning with CM from HOS cells potentiates the contraction capacities of BM‐MSCs (Fig. 2C). These results confirm the trans‐differentiation of BM‐MSCs towards a CAF‐like phenotype upon contact with tumour CM.

Therefore, to investigate whether the acquisition of this activated phenotype could enhance the pro‐tumourigenic activity of BM‐MSCs, we prepared CM either from BM‐MSCs maintained in St Med (CM BM‐MSCs ^St^) or stimulated with CM derived from HOS cells (CM BM‐MSCs ^HOS^) for 48 h. HOS cells, the cell line showing the highest invasion and migration potential (Fig. S3; Lauvrak *et al*., 2013; Ottaviano *et al*., 2010), were treated for 48 h with CM and evaluated for migration, invasion and transendothelial migration abilities. We found that both BM‐MSCs ^St^ and BM‐MSCs ^HOS^ potentiate the invasive behaviour of OS cells, suggesting an increase of OS malignancy induced by BM‐MSCs despite their activation levels (Figs 2D‐F and S4A‐C).

### 3.3 Cross‐talk between BM‐MSCs and OS guides OS cells towards an amoeboid cell motility

**Figure 3:**
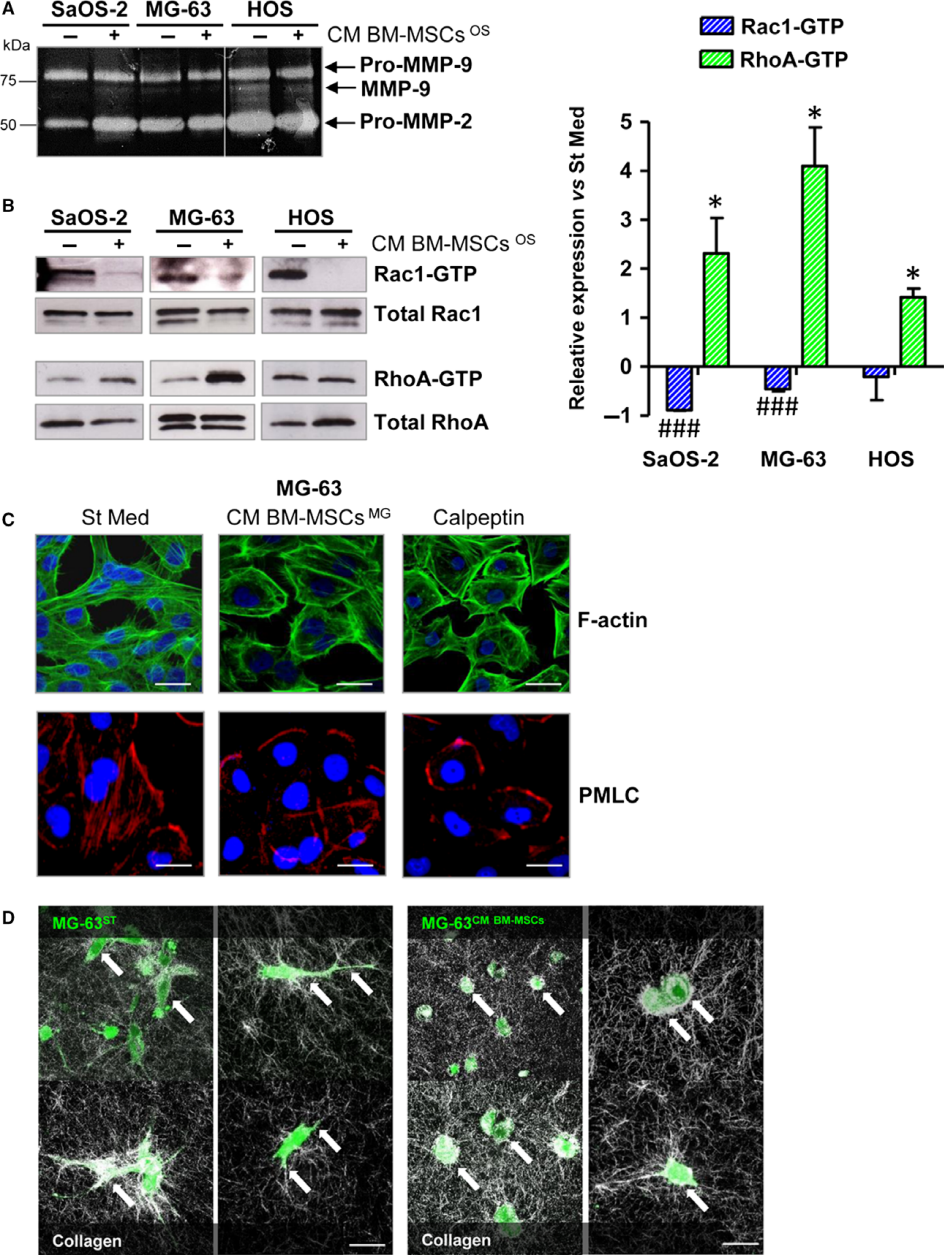
Cross‐talk between BM‐MSCs and OS cells promotes the acquisition of an amoeboid‐like motility in cancer cells. (A) Gelatin zymography of CM obtained from SaOS‐2, MG‐63 and HOS cells stimulated or not stimulated for 48 h with CM derived from BM‐MSCs activated by each OS cell line (CM BM‐MSCs ^OS^). The white line indicates the junction of two different gels. Image is representative of three independent experiments. (B) Representative images of pull‐down assay of Rac1 and RhoA GTPases (left panel) and related quantification (right panel). The assay was performed on OS cells grown for 48 h in CM from tumour‐activated BM‐MSCs (CM BM‐MSCs OS) or in starvation medium. Rac1‐GTP and RhoA‐GTP expression was normalized with respect to total Rac1 and RhoA in OS lysates. ^###^ *P *<* *0.001, Rac1‐GTP CM BM‐MSCs vs. Rac1‐GTP St Med. * *P *<* *0.05, RhoA‐GTP CM BM‐MSCs vs. RhoA‐GTP St Med. Results are presented as mean ± SD of three biological replicates. (C) Confocal analysis of F‐actin (FITC phalloidin) and P‐MLC staining in MG‐63 cells treated or not treated with CM BM‐MSCs ^MG^ ^−63^ for 48 h. Scale bar: 10 μm. The images are representative of three biological replicates with similar results. (D) Live imaging of MG‐63 cell migration in three‐dimensional collagen lattice. CFSE‐loaded MG‐63 cells were incorporated into the collagen matrix and monitored by confocal fluorescence‐reflection video microscopy. Tumour cells are visualized in green and the back‐scatter signal of the collagen I is shown in white. On the left, arrows indicate the point at which MG‐63, treated with ST medium, shows an elongated morphology. In MG‐63 cells treated for 48 h with CM from tumour‐activated BM‐MSCs (right), arrowheads indicate the rounded shape of the cells squeezing across collagen I fibres. Scale bar: 20 μm.

To investigate in depth the molecular mechanism leading to increased invasion potential of OS cells induced by BM‐MSCs, we decided to investigate the activity or expression of MMPs in OS cells conditioned with BM‐MSCs ^OS^ CM. As BM‐MSCs ^St^ and BM‐MSCs ^HOS^ showed a similar effect on OS migration abilities, we decided to use only CM derived from tumour‐activated BM‐MSCs in the following experiments to mimic *in vivo* conditions. We proved, by gelatin zymography, that all tumour cell lines secrete significant amounts of pro‐gelatinases MMP‐2 and MMP‐9 (Fig. 3A). HOS cells, the most aggressive OS cell line, secrete the highest levels of these MMPs; however, these do not increase following the treatment with CM BM‐MSCs ^HOS^. MG‐63 cells show a similar behaviour, whereas SaOS‐2 cells exhibit an increase in pro‐MMP‐2 following 48 h of incubation with CM from BM‐MSCs ^SaOS^. Next, we analysed the activation levels of RhoA GTPase, a key regulator of amoeboid migration, and Rac1 GTPase, which is required for mesenchymal motility (Sanz‐Moreno *et al*., 2008). The contact with CM derived from tumour‐activated BM‐MSCs induces a significant decrease of bound Rac1‐GTP with a parallel increase of bound RhoA‐GTP in OS cells. Notably, the CM treatment induces an approximately two‐fold increase in the RhoA/Rac1 ratio in all the OS cell lines tested (Fig. 3B, left and right panels). These data suggest a shift in the migration strategy of OS cells from a mesenchymal‐like to an amoeboid‐like motility. Moreover, confocal analysis shows that following the treatment with CM derived from tumour‐activated BM‐MSCs, MG‐63 cells acquire a rounded morphology and undergo a redistribution of actin fibres (Fig. 3C). In keeping with this, the active form of myosin light chain (P‐MLC) localizes in cortical rings, similar to cells treated with the RhoA activator, Calpeptin, whereas control cells (St Med) display an elongated morphology and actin‐myosin cytoskeleton organized in parallel bundles along the cytoplasm. The shift toward an amoeboid motility style is also supported by live imaging of MG‐63 cells in a three‐dimensional collagen lattice (Fig. 3D). When exposed to three‐dimensional matrices of type I collagen, untreated MG‐63 cells move through a spindle‐shaped mesenchymal and proteolytic stage. The conditioning of MG‐63 cells with CM from tumour‐activated BM‐MSCs promotes a shift to a rounded squeezing movement, independent of matrix degradation. Finally, in line with a shift toward an amoeboid‐like motility, OS cells stimulated by tumour‐activated BM‐MSCs display a lack of sensitivity to the treatment with the MMPs inhibitor Ilomastat in both invasion and transendothelial migration assays (Fig. S5A,B). Interestingly, we observed a significant increase in the levels of tissue inhibitor of MMP (TIMP)‐1 and ‐2 secretion in BM‐MSCs stimulated for 48 h with CM from HOS and MG‐63 cells (Fig. S6C). These data suggest that the reciprocal interplay between BM‐MSCs and cancer cells promotes tumour cell plasticity toward an amoeboid‐like phenotype.

### 3.4 GRO‐α, IL‐6, IL‐8 and MCP‐1 produced by BM‐MSCs determine the migration plasticity of OS cells

**Figure 4:**
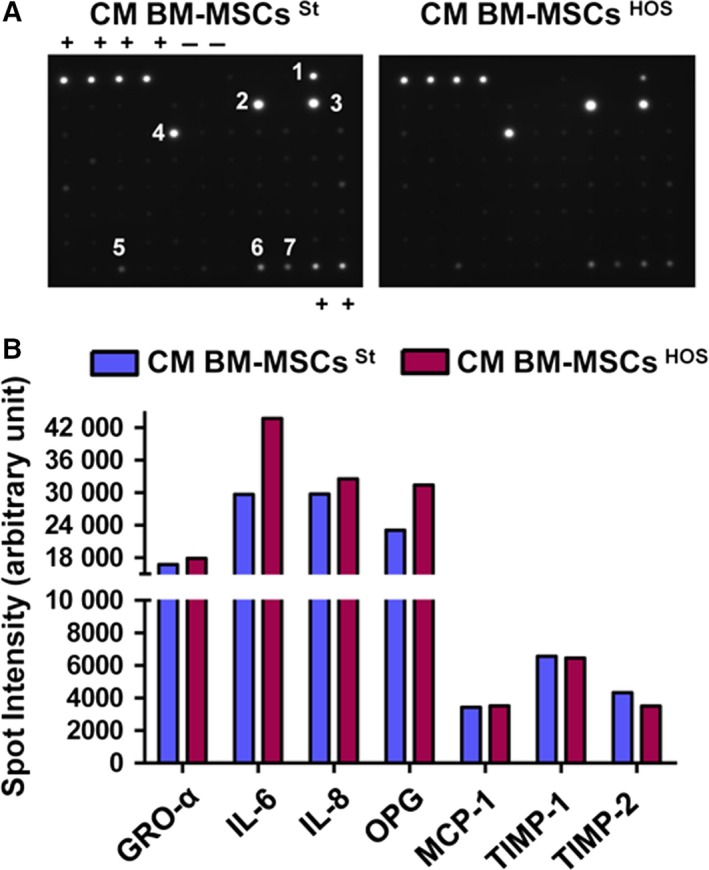
BM‐MSCs secrete a specific pattern of cytokines and growth factors. (A) CM from BM‐MSCs grown in St Med or HOS CM was collected and analysed with Human Cytokine Antibody Array according to manufacturer's protocol. C5 (+) positive controls; (−) negative controls; (1) GRO; (2) IL‐6; (3) IL‐8; (4) MCP‐1; (5) OPG; (6) TIMP‐1; (7) TIMP‐2. (B) Bar graph reporting the spot density quantified on each membrane with imagej. Expression values > 10 000 arbitrary units were set to identify a threshold for cytokines to be considered.

**Figure 5:**
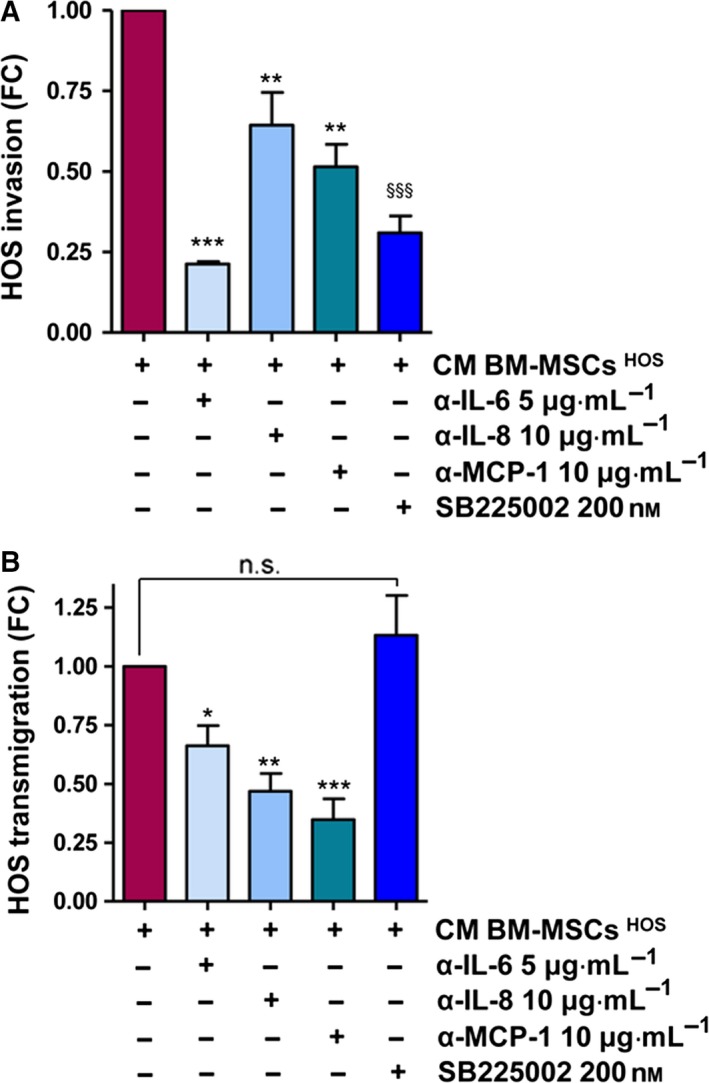
The pro‐tumourigenic activities of BM‐MSCs depend on GRO‐α, IL‐6, IL‐8 and MCP‐1 secretion. (A) Invasion assay of HOS cells treated with CM BM‐MSCs ^HOS^ supplemented or not supplemented with neutralizing antibodies against IL‐6 (5 μg·mL^−1^), IL‐8 (10 μg·mL^−1^) and MCP‐1 (10 μg·mL^−1^) and GRO‐α receptor inhibitor (SB 225002, 200 nm). Results are presented as mean ± SEM of three biological replicates. ** *P *<* *0.005 CM BM‐MSCs ^HOS^ vs. CM BM‐MSCs ^HOS^ + α‐IL‐8 and CM BM‐MSCs ^HOS^ vs. CM BM‐MSCs ^HOS^ + α‐MCP‐1; *** *P *<* *0.001, CM BM‐MSCs ^HOS^ vs. CM BM‐MSCs ^HOS^ + α‐IL‐6; ^§§§^ *P *<* *0.001, CM BM‐MSCs ^HOS^ vs. CM BM‐MSCs ^HOS^ + SB 225002. (B) Transendothelial migration assay of HOS cells treated as in (A) (mean ± SEM,* n *=* *3 biological replicates). * *P *<* *0.05, CM BM‐MSCs ^HOS^ vs. CM BM‐MSCs ^HOS^ + α‐IL‐6; ** *P *<* *0.005, CM BM‐MSCs ^HOS^ vs. CM BM‐MSCs ^HOS^ + α‐IL‐8; *** *P *<* *0.001, CM BM‐MSCs ^HOS^ vs. CM BM‐MSCs ^HOS^ + α‐MCP‐1.

To identify the soluble factors produced by BM‐MSCs responsible for changes induced in the style of motility of OS cells, we performed a cytokine assay of CM derived from unconditioned and tumour‐activated BM‐MSCs (Fig. 4A). The assay simultaneously detects 80 different cytokines and growth factors. Among these, we found detectable spots of GRO‐α, IL‐6, IL‐8, osteoprotegerin (OPG), a decoy receptor of RANKL, MCP‐1, TIMP‐1 and TIMP‐2. The relative bar plot is depicted in Fig. 4B. Expression values > 10 000 arbitrary units were set to identify a threshold for cytokines to be considered. To verify whether different OS cell lines share this pattern of cytokines and GFs, we stimulated BM‐MSCs isolated from two different donors with CM from both HOS and MG cells. Levels of GRO‐α, IL‐6, IL‐8 and MCP‐1 were measured by ELISA immunoassay (Table 1). As shown, we demonstrated that even if some differences in cytokines concentration are present at the basal level, most probably due to intra‐individual variability, the amount of these cytokines is similar between the two isolations. Furthermore, the increase of cytokine expression following conditioning with CM from both OS cell lines follows exactly the same trend, suggesting the crucial role of these cytokines in promoting aggressiveness of OS cell lines. To validate the role of these factors in modulating the migration abilities of cancer cells, HOS cells stimulated with CM BM‐MSCs ^HOS^ were treated or not treated with neutralizing antibodies against IL‐6, IL‐8, MCP‐1 and SB225002, and then analysed for invasion and transmigration. As depicted in Figs 5A and S7A, the invasion of cancer cells is strongly dependent on this pattern of cytokines. In particular, IL‐6 and GRO‐α blockade drastically impairs HOS cell invasion. On the other hand, HOS transmigration is significantly affected by IL‐8 and MCP‐1 inhibition, but blocking GRO‐α signalling does not affect the migration of cancer cells across the endothelial monolayer (Figs 5B and S6B). To evaluate the specificity of treatment, normal mouse IgG was used as control (Fig. S6C). As the combined treatment with all the inhibitors leads to a strong decrease of cell viability (almost 60%) following 24 h of incubation with inhibitors, we excluded this condition from the experimental settings (Fig. S6D).

**Table 1:** BM‐MSCs isolated from two different healthy donors (BM‐MSC 1 and 2) were grown to confluence, then serum‐starved (St Med) or stimulated with CM from HOS and MG‐63 cells for 48 h. The media were replaced with St Med for another 24 h, then collected, clarified by centrifugation and analysed by ELISA immunoassay for GRO‐α, IL‐6, IL‐8 and MCP‐1. The results are the mean ± SD of three technical replicates

	BM‐MSC 1			BM‐MSC 2		
	St Med	CM HOS	CM MG‐63	St Med	CM HOS	CM MG‐63
GRO‐α	7 pg mL^−1^ ± 0.2	11 pg mL^−1^ ± 1	13 pg mL^−1^ ± 1	6 pg mL^−1^ ± 0.1	8 pg mL^−1^ ± 0.3	28 pg mL^−1^ ± 9
IL‐6	11 pg mL^−1^ ± 3	24 pg mL^−1^ ± 5	700 pg mL^−1^ ± 190	79 pg mL^−1^ ± 13	223 pg mL^−1^ ± 13	6557 pg mL^−1^ ± 223
IL‐8	6 pg mL^−1^ ± 0.3	50 pg mL^−1^ ± 6	177 pg mL^−1^ ± 20	10 pg mL^−1^ ± 2	42 pg mL^−1^ ± 12	382 pg mL^−1^ ± 73
MCP‐1	89 pg mL^−1^ ± 21	598 pg mL^−1^ ± 151	647 pg mL^−1^ ± 120	783.9 pg mL^−1^ ± 151	1104.9 pg mL^−1^ ± 126	991.8 pg mL^−1^ ± 177

### 3.5 Reciprocal interplay between BM‐MSCs and OS cells stimulates *in vitro* angiogenesis

**Figure 6:**
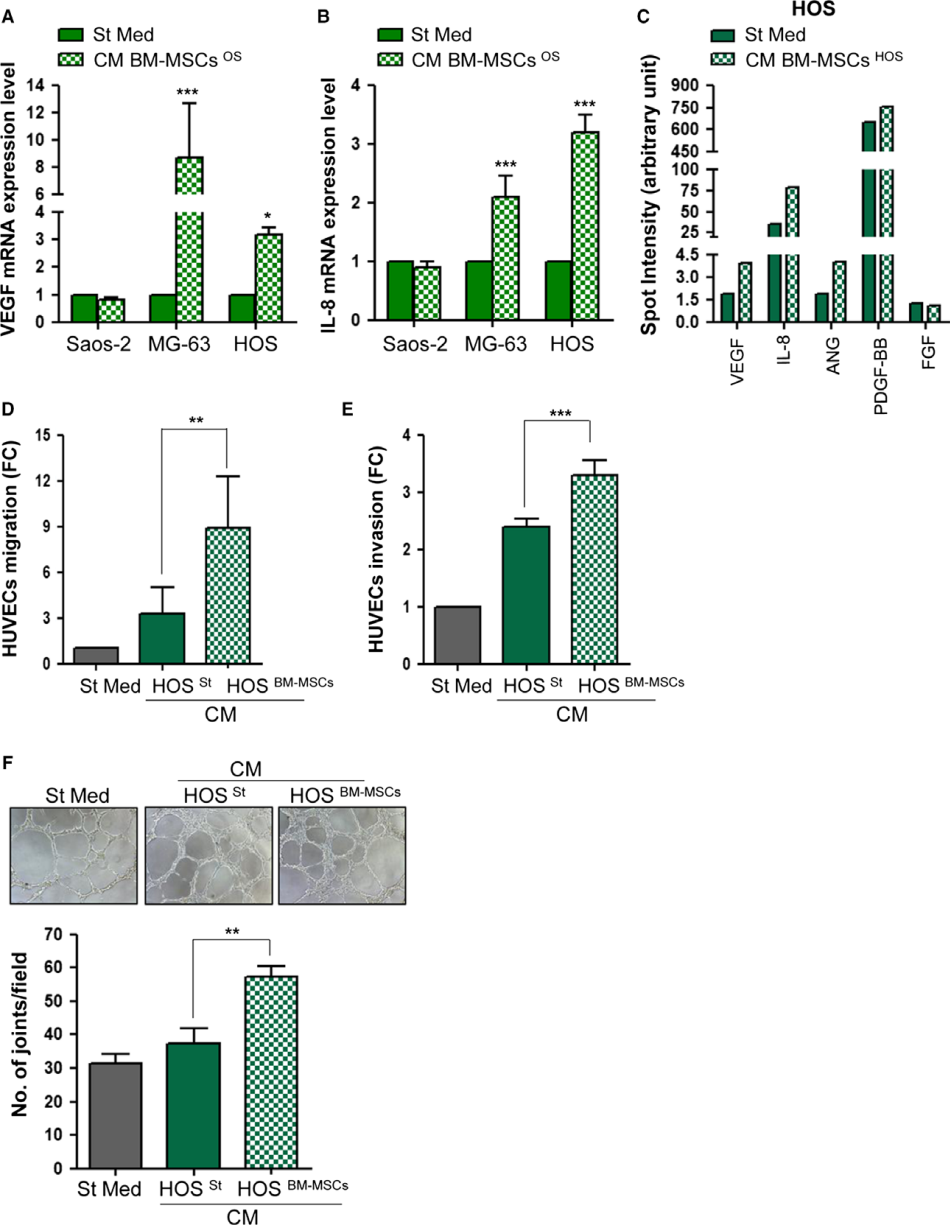
Cross‐talk between BM‐MSCs and OS cells stimulates the recruitment and activation of endothelial cells. (A,B) Quantitative RT‐PCR of *VEGF* and *IL‐8* mRNA expression in OS cells maintained in St Med or CM BM‐MSCs ^OS^ for 48 h. Results are presented as mean ± SD of three biological replicates. * *P *<* *0.05 vs. St Med; *** *P *<* *0.001 vs. St Med. (C) Semi‐quantitative detection of pro‐angiogenic factors in CM derived from HOS cells stimulated with CM from tumour‐activated BM‐MSCs in comparison with CM derived from BM‐MSCs maintained in St Med for 48 h. CM were collected and analysed with Human Cytokine Antibody Array according to manufacturer's protocol reported in Material and Methods. (D) Migration assay of HUVEC cells towards CM derived from HOS ^St^ or HOS ^BM^ ^‐^ ^MSC^ ^s^. Results are mean ± SEM from three independent experiments. ** *P *<* *0.05 vs. St Med; *** *P *<* *0.001 vs. St Med. (E) Invasion assay of HUVEC cells treated as in (D). Results are expressed as mean ± SEM of three biological replicates.** *P *<* *0.05 CM HOS ^BM^ ^‐^ ^MSC^ ^s^ vs. CM HOS ^St^; *** *P *<* *0.001 CM HOS^BM^ ^‐^ ^MSC^ ^s^ vs. CM HOS ^St^. (F) Capillary morphogenesis assay of HUVECs incubated in CM from HOS ^St^ or HOS ^BM^ ^‐^ ^MSC^. Cord formation was examined after 6 h at 37 °C with optical microscope. The total number of junctions is presented as mean ± SEM of three randomly chosen fields for each experimental condition of three biological replicates performed in technical duplicate. ** *P *<* *0.05 vs. St Med.

The formation of new vessels is a key prerequisite to ensure the metastatic dissemination of cancer cells. Moreover, as the cross‐talk between BM‐MSCs and OS cells greatly affects their transendothelial migration capacities, we decided to investigate whether this interplay could also influence the activation of endothelial cells. First, quantitative RT‐PCR analysis revealed an increase in the mRNA levels of *VEGF* and *IL*‐*8*, two of the major activators of angiogenesis, in HOS or MG‐63 cells maintained for 48 h in St Med or CM from BM‐MSCs conditioned by OS cells (Fig. 6A,B). To confirm the expression of VEGF and IL‐8 at protein level, measurements were taken using a cytokine array a panel of pro‐angiogenic factors in CM derived from HOS cells maintained in St Med or stimulated with CM from tumour‐activated BM‐MSCs for 48 h. As shown in Fig. 6C, HOS cells activated with CM from BM‐MSCs secrete higher amounts of VEGF, IL‐8, angiopoietin (ANG) and platelet‐derived growth factor BB (PDGF‐BB). We then evaluated the ability of HOS cells to recruit endothelial cells in both chemoattraction and invasion assays. To this end, HUVECs were allowed to migrate toward or to invade CM HOS ^St^ or CM HOS ^BM‐MSCs^. As shown in Figs 5(C,D) and S7(A,B), the interplay between BM‐MSCs and HOS cells strongly potentiates both chemoattraction and invasiveness of endothelial cells. Similarly, we demonstrated that this cross‐talk is crucial to stimulate the capillary network formation *in vitro,* already appreciable following 6 h of treatment (Fig. 6F). These results strongly support the idea that in OS stroma, BM‐MSCs and cancer cells co‐operate to promote tumour vascularization, a central phenomenon for cancer growth and metastasis.

## 4 Discussion

The tumour microenvironment is a complex and dynamic milieu consisting of cells, signalling molecules and ECM that supports tumour growth and progression. Here, we investigated the effects induced by the cross‐talk between BM‐MSCs and OS cells on tumour malignancy. We found that BM‐MSCs are efficiently recruited by three different OS cell lines (SaOS‐2, MG‐63 and HOS) and, for the first time, we identified MCP‐1, GRO‐α and TGF‐β‐1 as key molecules in promoting this migration. Moreover, we showed that following contact with CM from OS cells, BM‐MSCs are stimulated to trans‐differentiate into CAF‐like cells. The interaction between BM‐MSCs and OS cells is bidirectional: the mesenchymal stroma activated by tumour cells secrete higher levels of IL‐6, IL‐8, GRO‐α and MCP‐1 in the tumour microenvironment. OS cells replay the changes of the microenvironment through a MAT, further enhancing their invasiveness and transendothelial migration abilities. Moreover, the interplay between BM‐MSCs and OS cells significantly affects the activation of endothelial cells in terms of invasion, migration and capacity to form a capillary network. Here, we characterized the response of three different OS cell lines. These cells significantly diverge from each other in genetic alteration, morphology, aggressiveness and proliferation kinetics (Lauvrak *et al*., 2013; Mohseny *et al*., 2011). In particular, SaOS‐2 cells show a phenotype very close to that of normal mesenchymal precursors, whereas HOS cells, which are extremely aggressive, display a smaller and hexagonal morphology, similar to epithelial cells. The MG‐63 cells have an intermediate phenotype between the other two OS cell lines. Following contact with CM from BM‐MSCs, SaOS‐2 cells seem to behave in a different manner compared with MG‐63 and HOS cells with regard to cell migration and MMP expression, as well as secretion of pro‐angiogenic factors. This is probably due their striking mesenchymal phenotype and a basal aggressiveness that is lower than that of the other two lines. However, SaOS‐2 cells showed the most remarkable reduction of the active form of Rac‐1, underlining that the mesenchymal stromal compartment can strongly affect the tumour cell phenotype and thus the OS evolution.

It is now well established that several chemokines and growth factors are involved in the mobilization of BM‐MSCs from the bone to the stroma of different tumours, such as glioma, breast, prostate, ovarian, pancreatic and lung carcinoma (Barcellos‐de‐Souza *et al*., 2013; Spaeth *et al*., 2008; Xu *et al*., 2009). Notably, in this study, we show that OS‐dependent recruitment of BM‐MSCs is supported by a specific pattern of cytokines including MCP‐1, GRO‐α and TGF‐β1. Moreover, we excluded the involvement of CXCR4/SDF‐1 axis in this migration. In agreement with our results, several reports indicate that BM‐MSCs secrete high levels of SDF‐1 but express low levels of CXCR4, whereas OS cells release small amounts of SDF‐1 but express elevated levels of CXCR4, crucial to promote the metastatic spread of the tumour to the lung (Perissinotto *et al*., 2005; Ponte *et al*., 2007; Yu *et al*., 2015).

Several studies hae shown that, once engrafted into the tumour microenvironment, BM‐MSCs are able directly to aid tumour growth and progression. Here, we show that BM‐MSCs promote both invasion and transendothelial migration of OS cells, suggesting an increase in the metastatic potential of cancer cells. This observation is in agreement with data showing that cytokines secreted by BM‐MSCs not previously in contact with cancer cells, can directly support proliferation and migration of tumour cells (Karnoub *et al*., 2007; Martin *et al*., 2010; Tsai *et al*., 2011; Xu *et al*., 2009).

It is well known that, following cytokine and growth factor stimulation, BM‐MSCs trans‐differentiate into different stromal components, leading to promotion of tumour malignancy. Herein, we have proved that following contact with CM from OS cells, BM‐MSCs trans‐differentiate into α‐SMA‐expressing fibroblasts, normally identified as CAFs. Surprisingly, we noticed that activated CAF‐like BM‐MSCs induce a similar increase in the migratory/invasive abilities of OS cells with respect to non‐activated BM‐MSCs. Most probably because we tested only standard conditions (without hypoxia or acidosis), and also because OS is a mesenchymal tumour with a basal, very high aggressiveness, as demonstrated by the early development of pulmonary metastasis, this resulted in high patient mortality. Thus, it is very hard to further enhance this malignant behaviour.

We have now demonstrated that recruited BM‐MSCs promote a shift toward an amoeboid phenotype in OS cells. As a consequence, OS cells potentiate their invasion and transendothelial migration, increasing their metastatic potential. In keeping with our results, Cortini *et al*. (2016) have recently demonstrated that IL‐6 secreted by the mesenchymal stroma is essential to promote OS stemness and migratory potential. In line with this, a previous work by our group indicated that CAFs co‐operate with endothelial progenitor cells to engage a clear MAT in prostate cancer cells (Giannoni *et al*., 2013). This shift in motility style is crucial to promote cancer cell adhesion to endothelium and transendothelial migration. MAT has been described as an essential adaptive programme conferring significant advantages during the metastatic dissemination. To date, very little is known about the molecular mechanisms that govern this transition. It is widely accepted that growth factors and cytokines released by either tumour cells themselves or stromal cells not only control and direct the migration routes of tumour cells, but also modulate their plasticity, invasiveness and metastatic dissemination (Odenthal *et al*., 2016). It has been recently proved that metastatic sarcoma cells show an up‐regulation of RhoA/ROCK signalling compared with parental non‐metastatic cells (Belgiovine *et al*., 2010; Rösel *et al*., 2008; Zucchini *et al*., 2014). In the present study, we showed that BM‐MSCs promote MAT in OS cells through the secretion of a specific pattern of cytokines: GRO‐α, IL‐6, IL‐8 and MCP‐1. Using specific inhibitors, we demonstrated that each of these cytokines, particularly IL‐6 and GRO‐α, modulates the invasive behaviour of tumour cells, whereas the mechanism of transendothelial migration is completely independent of GRO‐α/CXCR2 signalling. In keeping with our results, it has been reported that in squamous carcinoma cells, melanoma and stromal fibroblasts, IL‐6 pathway activates ROCK and generates a high level of actomyosin contractility (Sanz‐Moreno *et al*., 2011). Moreover, it has been shown that the activation of IL‐8 signalling pathways leads to RhoA activation and actin stress fibre formation in cancer and endothelial cells (Schraufstatter *et al*., 2001; Waugh and Wilson, 2008). Finally, several studies have indicated that MCP‐1 secreted by tumoural and stromal cells induces transendothelial migration of T cells, monocytes, smooth muscle cells and adult neural stem cells (Cai *et al*., 1996; Ma *et al*., 2007; Widera *et al*., 2004). Interestingly, we showed that the pattern of cytokines secreted by both BM‐MSCs and OS cells is very similar, confirming the common mesenchymal origin of these two cell types (Kansara *et al*., 2014; Meyers *et al*., 2011). Most likely, the recruitment of BM‐MSCs to the tumour site promotes a local increase of the cytokines which are already produced by tumour cells themselves. Thus, both cell populations concurrently contribute to the generation of a milieu enriched in cytokines, which stimulates in an additive manner the migratory and invasive properties of OS cells.

Finally, we stress that the cross‐talk between BM‐MSCs and OS cells on the one hand stimulates the secretion of pro‐angiogenic factors in tumour cells, and on the other stimulates both the recruitment and the invasion of endothelial cells, as well as their capacity to form *in vitro* tubular‐like structures. The formation of new vessels combined with increased transendothelial migration of OS cells are additive events concurrently promoting the metastatic dissemination of cancer cells.

Our data indicate that the recruitment of BM‐MSCs into the OS stroma is a crucial event to promote tumour progression. Therefore, both recruitment of BM‐MSCs to the OS site and cytokine‐induced MAT of tumour cells represent innovative targets to test *in vivo* OS models to design innovative therapeutic approaches aiming to hinder the metastatic dissemination of OS cells.

## 5 Conclusions

Our data indicate that the recruitment of BM‐MSCs into the OS stroma is a crucial event to promote tumour progression. Indeed, once in contact with tumour cells, BM‐MSCs trans‐differentiate into CAFs, strongly increasing GRO‐α, MCP‐1, IL‐6 and IL‐8 levels in the tumour microenvironment. These cytokines are crucial to induce a MAT in OS cells, with a consequent rise of their migration abilities, invasiveness and transendothelial migration. Moreover, in response to these microenvironmental changes, tumour cells significantly increase their capacity to induce tumour *de novo* angiogenesis, a key step to ensure the metastatic dissemination of cancer cells. Therefore, our results include both recruitment of BM‐MSCs to the OS site and their transendothelial migration due to MAT as innovative targets to test *in vivo* OS models in order to design new therapeutic approaches aiming to impair the metastatic dissemination of OS cells.

## Author contributions

LP and MLT designed and performed the experiments. VG, ML, VB, LP and FB contributed reagents/materials/analysis tools and performed BM‐MSC characterization. PC contributed to the conception, design and overall project management. LP, MLT and PC wrote the manuscript.

## Supporting information


**Fig. S1.** (A,B) Representative images of BM‐MSC chemotaxis toward CM from OS cells in the presence or absence of CXCR4 inhibitor 20 μg·mL^−1^. (C) 3.5 × 10^5^ BM‐MSCs were serum‐starved for 24 h in the presence of α‐CXCR4 or normal mouse IgG (20 μg·mL^−1^), then allowed to migrate overnight toward CM from OS cells. (D) Representative images of BM‐MSC migration in the presence of specific inhibitors of MCP‐1, GRO‐α and TGF‐β (see Material and Methods for more details). (E) 3.5 × 10^5^ BM‐MSCs were serum‐starved for 24 h and allowed to migrate toward CM from MG‐63 cells supplemented or not supplemented with neutralizing antibodies against MCP‐1 or with normal mouse IgG.; *** *P *<* *0.001 vs. St Med. (F) 3.5 × 10^5^ BM‐MSCs were starved overnight in the presence or absence of 200 nm SB225002, 100 μg·mL^−1^ TβR blk and both inhibitors (Combo). Cells were then detached, centrifuged, resuspended in Muse™ Count and Viability buffer (1 × 10^5^ cells·mL^−1^) and cell viability assessed with Muse® Cell Analyzer according to manufacturer's instructions.

**Fig. S2.** Representative images of OS cell invasion (A) and transendothelial migration (B) (see Results section for more details).

**Fig. S3.** 1.5 × 10^5^ OS cells (Saos‐2, MG‐63 and HOS) were starved overnight and allowed to migrate for 16 h toward complete medium (FBS 10%). Mean ± SEM of two biological replicates performed in triplicate.

**Fig. S4.** Representative images of HOS cell migration (A), invasion (B) and transendothelial migration (C) (see Results section for more details).

**Fig. S5.** OS cells were cultured for 48 h in St Med or CM derived from tumour‐activated BM‐MSCs (CM BM‐MSCs ^OS^) supplemented or not supplemented with MMP inhibitor Ilomastat, 50 μm. The invasion (A) or transendothelial migration (B) was evaluated by counting migrating cells in four randomly chosen fields (mean ± SEM, *n *=* *3 biological replicates). * *P *<* *0.05 St Med vs. St Med + Ilo; ** *P *<* *0.01 St Med vs. CM BM‐MSCs ^OS^; *** *P *<* *0.001 St Med vs. CM BM‐MSCs ^OS^; # *P *<* *0.05 St Med vs. CM BM‐MSCs ^OS^ + Ilo; ## *P *<* *0.01 St Med vs. CM BM‐MSCs ^OS^ + Ilo; ### *P *<* *0.001 St Med vs. CM BM‐MSCs ^OS^ + Ilo. (C) 3.5 × 10^5^ BM‐MSCs were starved (St Med) or conditioned with CM from HOS and MG‐63 cells for 48 h. BM‐MSCs were then starved for a further 24 h and media were collected, centrifuged and analysed by ELISA for quantification of TIMP‐1 and ‐2. Results are expressed as mean ± SD of three biological replicates. * *P *<* *0.001 vs. St Med.

**Fig. S6.** Representative images of HOS invasion (A) and transendothelial migration (B) in the presence of specific inhibitors of IL‐6, IL‐8, MCP‐1 and GRO‐α activity (see Results section for more details). (C) To evaluate whether the treatment with neutralizing antibodies could basically affect the migration abilities of cancer cells, 5 × 10^4^ HOS cells were treated for 48 h with CM from tumour‐activated BM‐MSCs in the presence or absence of normal mouse IgG 5 and 10 μg·mL^−1^. The mock antibodies did not significantly change HOS invasion. (D) 3.5 × 10^5^ HOS cells were maintained for 24 h in starvation medium (St Med) or in CM from tumour‐activated BM‐MSCs (CM BM‐MSCs ^HOS^) in the presence or absence of neutralizing antibodies against IL‐6 (5 μg·mL^−1^), IL‐8 (10 μg·mL^−1^), MCP‐1 (10 μg·mL^−1^), SB225002 (200 nm) and all inhibitors (Combo). Cells were then detached, centrifuged, resuspended in Muse™ Count and Viability buffer (1 × 10^5^ cells·mL^−1^) and assessed with Muse® Cell Analyzer according to the manufacturer's instructions.

**Fig. S7.** Representative images of HUVEC migration (A) and invasion (see Results section for more details).
