# Supplementary figures and images for: Bone marrow‐derived mesenchymal stem cells promote invasiveness and transendothelial migration of osteosarcoma cells via a mesenchymal to amoeboid transition
Source: Mol Oncol. 2018 Mar 31;12(5):659–76. doi: 10.1002/1878-0261.12189 (PMC5928379; doi:10.1002/1878-0261.12189)

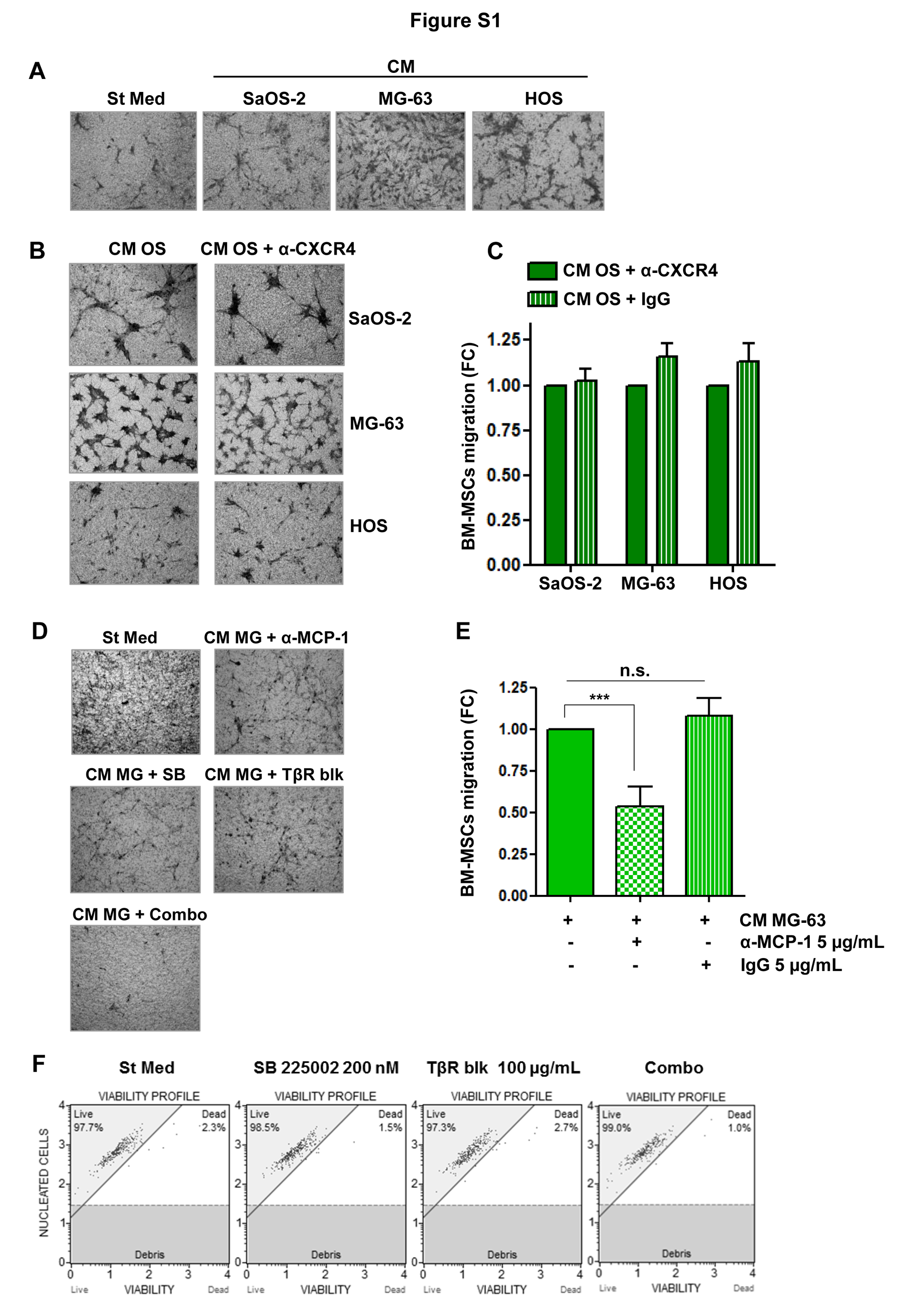

Supplement: Supplementary file 1 — Fig. S1. (A,B) Representative images of BM‐MSC chemotaxis toward CM from OS cells in the presence or absence of CXCR4 inhibitor 20 μg·mL−1. (C) 3.5 × 10^5^ BM‐MSCs were serum‐starved for 24 h in the presence of α‐CXCR4 or normal mouse IgG (20 μg·mL−1), then allowed to migrate overnight toward CM from OS cells. (D) Representative images of BM‐MSC migration in the presence of specific inhibitors of MCP‐1, GRO‐α and TGF‐β (see Material and Methods for more details). (E) 3.5 × 10^5^ BM‐MSCs were serum‐starved for 24 h and allowed to migrate toward CM from MG‐63 cells supplemented or not supplemented with neutralizing antibodies against MCP‐1 or with normal mouse IgG.; *** P < 0.001 vs. St Med. (F) 3.5 × 10^5^ BM‐MSCs were starved overnight in the presence or absence of 200 nm SB225002, 100 μg·mL−1 TβR blk and both inhibitors (Combo). Cells were then detached, centrifuged, resuspended in Muse™ Count and Viability buffer (1 × 10^5^ cells·mL−1) and cell viability assessed with Muse® Cell Analyzer according to manufacturer's instructions. [file MOL2-12-659-s001.png]

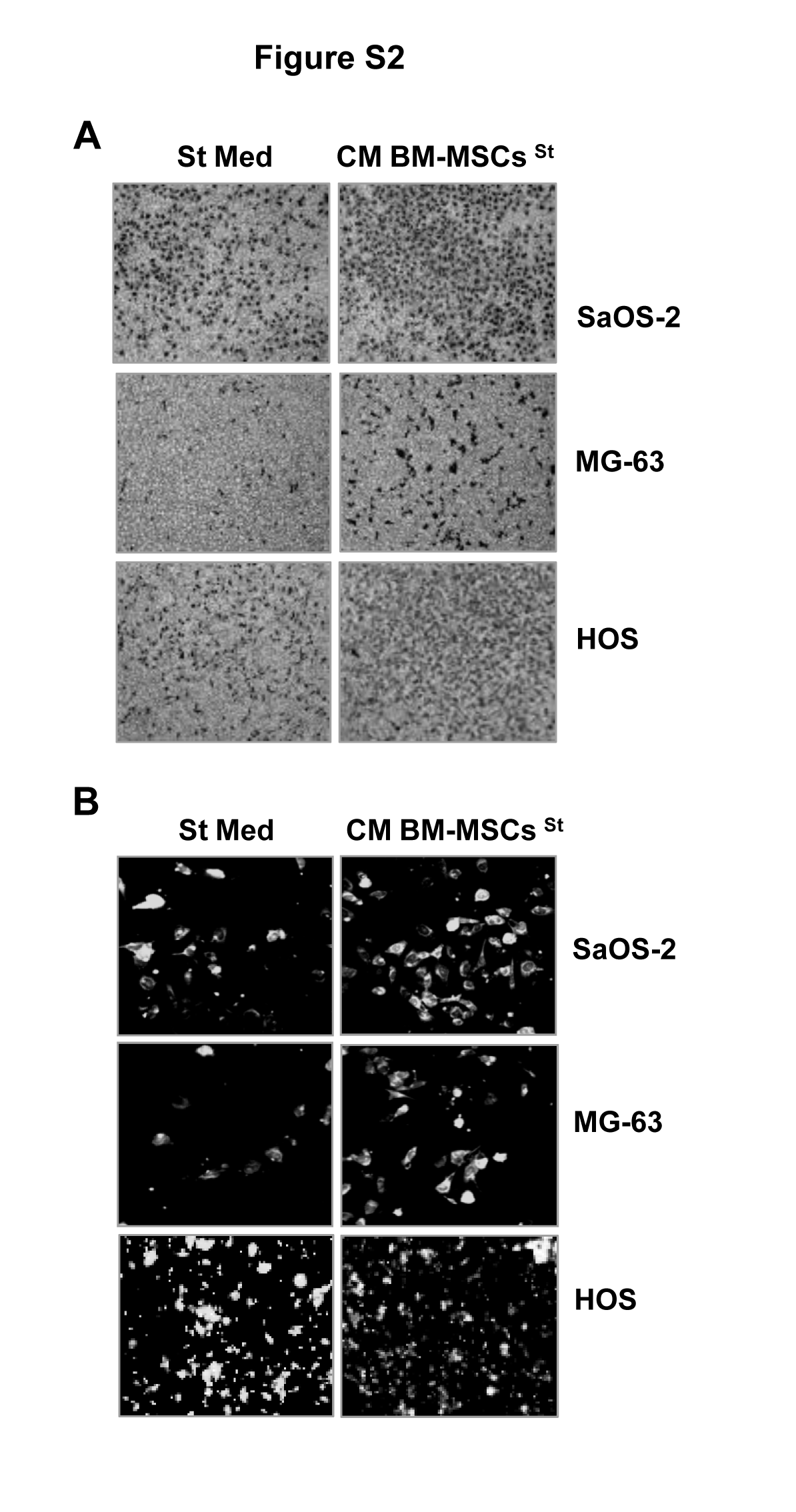

Supplement: Supplementary file 2 — Fig. S2. Representative images of OS cell invasion (A) and transendothelial migration (B) (see Results section for more details). [file MOL2-12-659-s002.png]

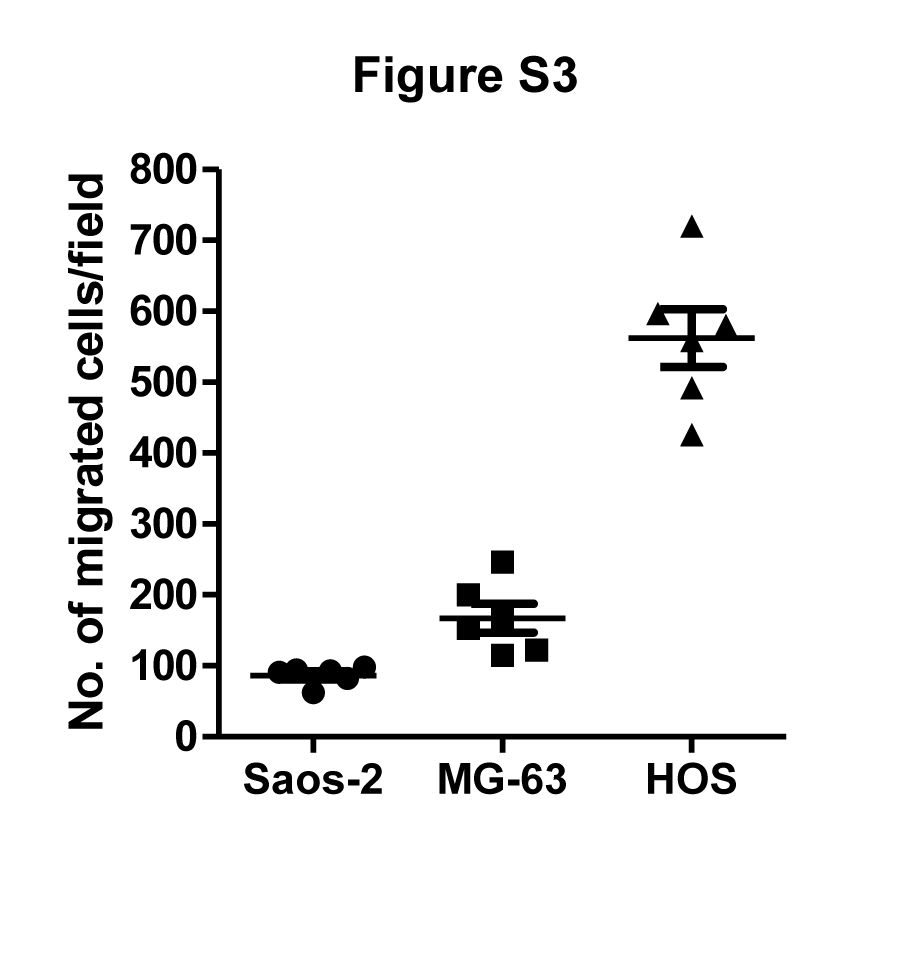

Supplement: Supplementary file 3 — Fig. S3. 1.5 × 10^5^ OS cells (Saos‐2, MG‐63 and HOS) were starved overnight and allowed to migrate for 16 h toward complete medium (FBS 10%). Mean ± SEM of two biological replicates performed in triplicate. [file MOL2-12-659-s003.png]

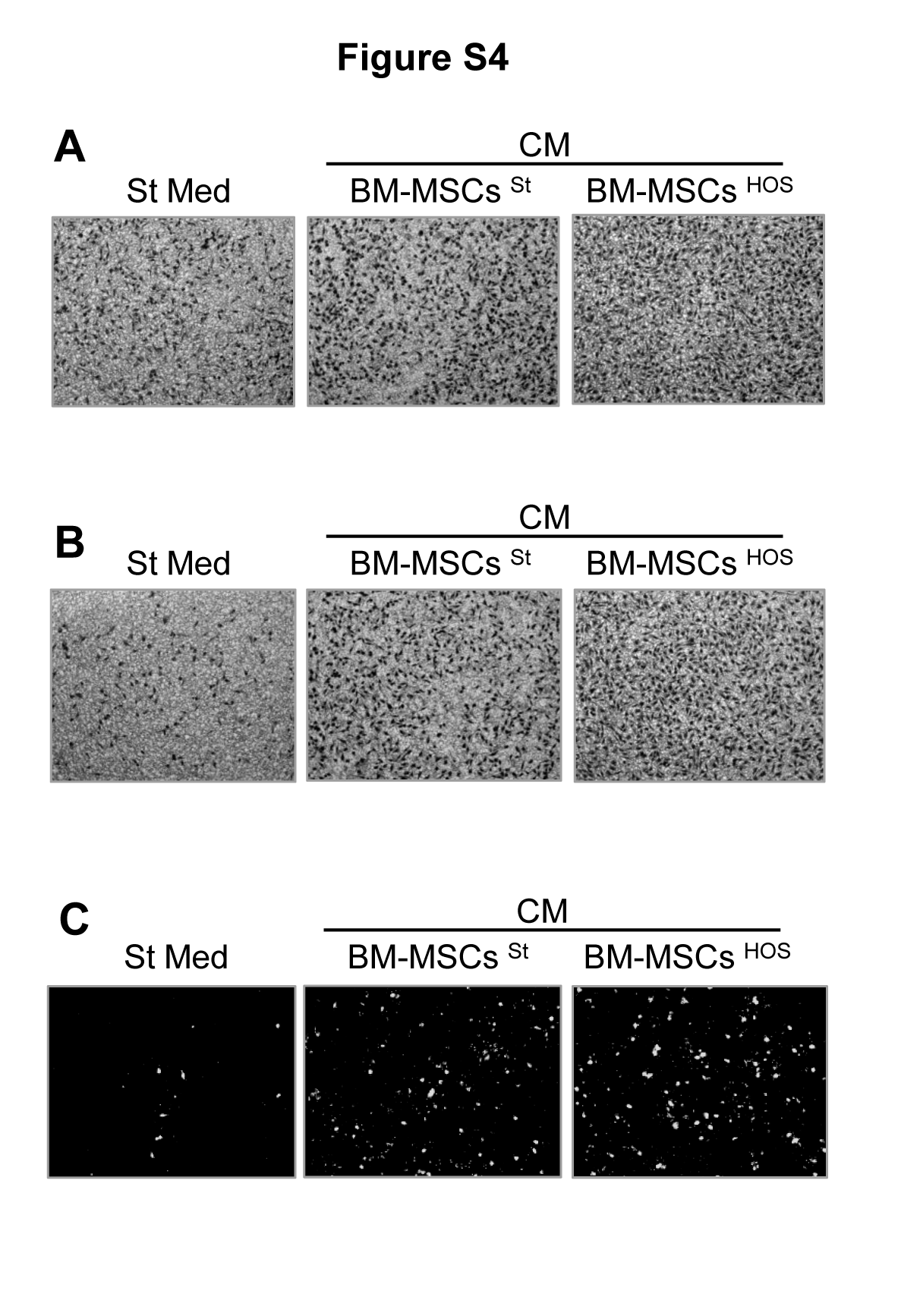

Supplement: Supplementary file 4 — Fig. S4. Representative images of HOS cell migration (A), invasion (B) and transendothelial migration (C) (see Results section for more details). [file MOL2-12-659-s004.png]

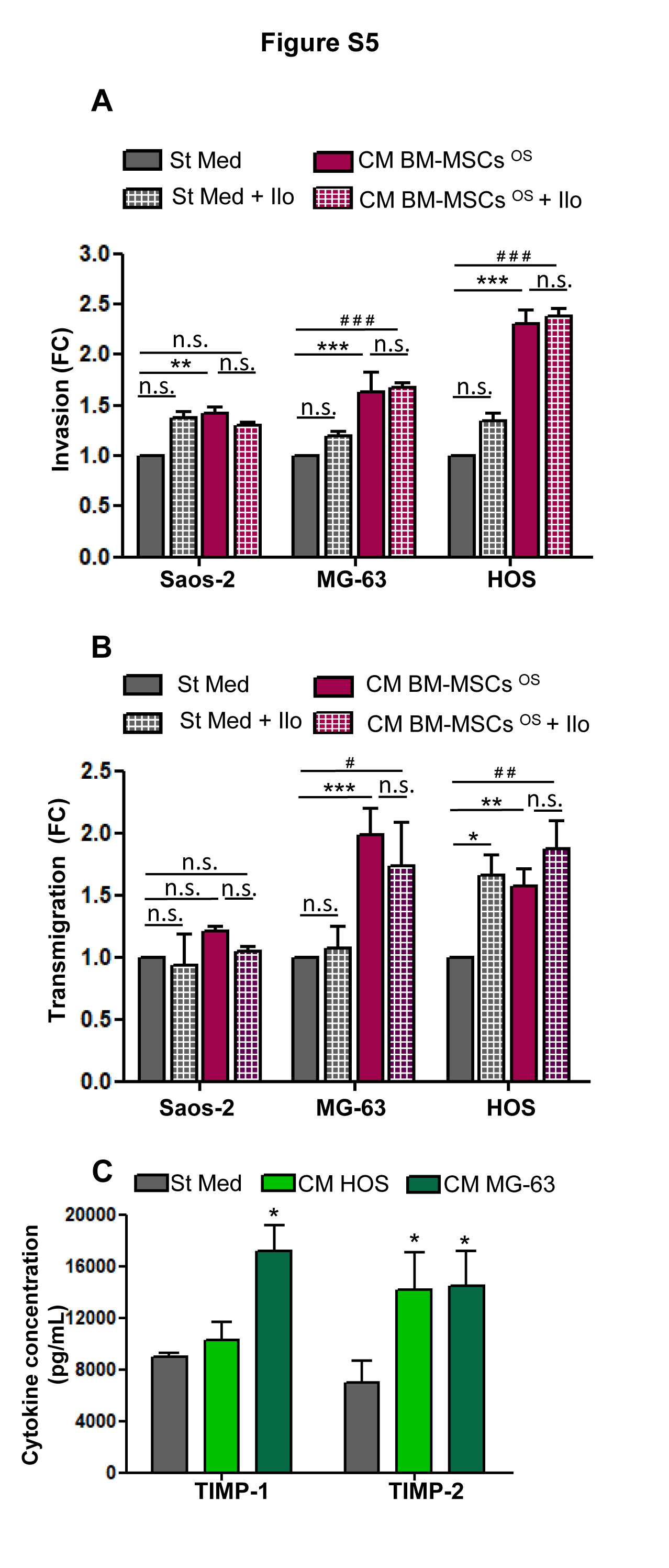

Supplement: Supplementary file 5 — Fig. S5. OS cells were cultured for 48 h in St Med or CM derived from tumour‐activated BM‐MSCs (CM BM‐MSCs OS) supplemented or not supplemented with MMP inhibitor Ilomastat, 50 μm. The invasion (A) or transendothelial migration (B) was evaluated by counting migrating cells in four randomly chosen fields (mean ± SEM, n = 3 biological replicates). * P < 0.05 St Med vs. St Med + Ilo; ** P < 0.01 St Med vs. CM BM‐MSCs OS; *** P < 0.001 St Med vs. CM BM‐MSCs OS; # P < 0.05 St Med vs. CM BM‐MSCs OS + Ilo; ## P < 0.01 St Med vs. CM BM‐MSCs OS + Ilo; ### P < 0.001 St Med vs. CM BM‐MSCs OS + Ilo. (C) 3.5 × 10^5^ BM‐MSCs were starved (St Med) or conditioned with CM from HOS and MG‐63 cells for 48 h. BM‐MSCs were then starved for a further 24 h and media were collected, centrifuged and analysed by ELISA for quantification of TIMP‐1 and ‐2. Results are expressed as mean ± SD of three biological replicates. * P < 0.001 vs. St Med. [file MOL2-12-659-s005.png]

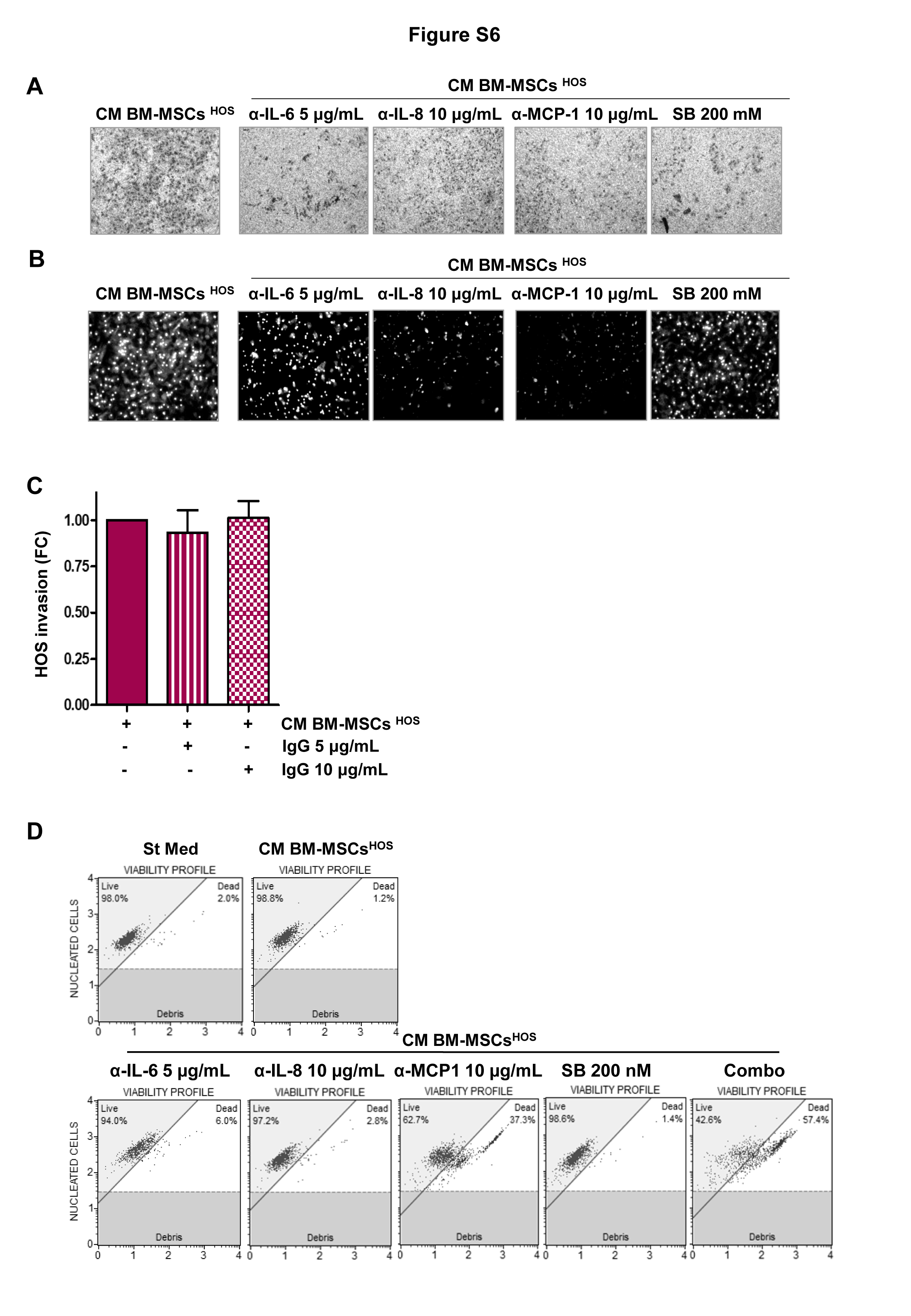

Supplement: Supplementary file 6 — Fig. S6. Representative images of HOS invasion (A) and transendothelial migration (B) in the presence of specific inhibitors of IL‐6, IL‐8, MCP‐1 and GRO‐α activity (see Results section for more details). (C) To evaluate whether the treatment with neutralizing antibodies could basically affect the migration abilities of cancer cells, 5 × 10^4^ HOS cells were treated for 48 h with CM from tumour‐activated BM‐MSCs in the presence or absence of normal mouse IgG 5 and 10 μg·mL−1. The mock antibodies did not significantly change HOS invasion. (D) 3.5 × 10^5^ HOS cells were maintained for 24 h in starvation medium (St Med) or in CM from tumour‐activated BM‐MSCs (CM BM‐MSCs HOS) in the presence or absence of neutralizing antibodies against IL‐6 (5 μg·mL−1), IL‐8 (10 μg·mL−1), MCP‐1 (10 μg·mL−1), SB225002 (200 nm) and all inhibitors (Combo). Cells were then detached, centrifuged, resuspended in Muse™ Count and Viability buffer (1 × 10^5^ cells·mL−1) and assessed with Muse® Cell Analyzer according to the manufacturer's instructions. [file MOL2-12-659-s006.png]

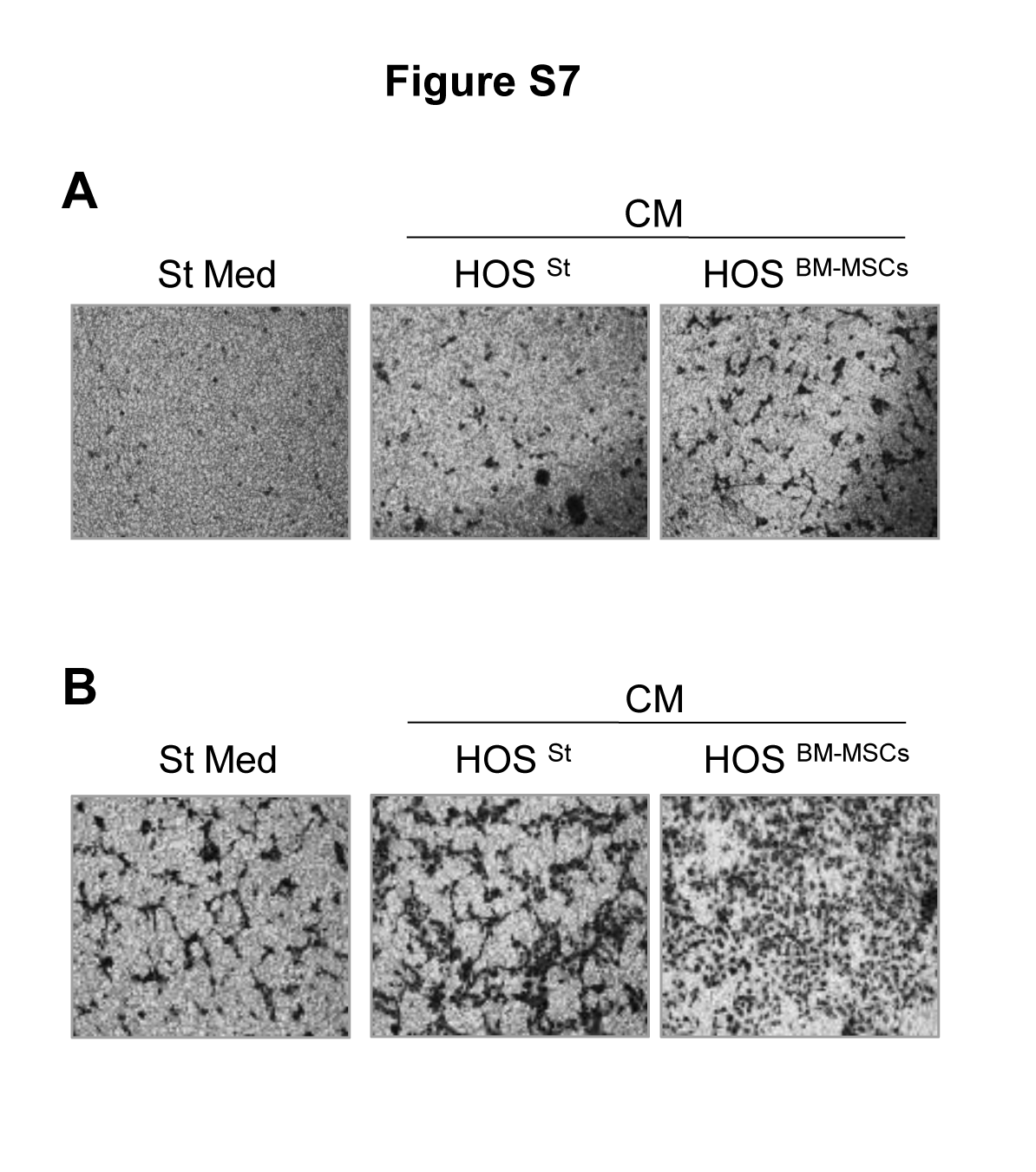

Supplement: Supplementary file 7 — Fig. S7. Representative images of HUVEC migration (A) and invasion (see Results section for more details). [file MOL2-12-659-s007.png]
